# Potent anticancer activity of phenolic-enriched extracts from endemic plants supported by *in vitro* pharmacological analyses and *in silico* molecular docking

**DOI:** 10.1007/s00114-026-02079-2

**Published:** 2026-02-16

**Authors:** Bugrahan Emsen, Mustafa Cicek, Burak Surmen, Hacer Sibel Karapinar

**Affiliations:** 1 Department of Biology, Kamil Özdağ Faculty of Science, Karamanoğlu Mehmetbey University, Karaman, Türkiye; 2 Department of Chemistry, Kamil Özdağ Faculty of Science, Karamanoğlu Mehmetbey University, Karaman, Türkiye

**Keywords:** Bioactive compounds, Pharmaceutical potential, Phytochemicals, Phytotherapy, Polyphenols

## Abstract

In the present study, methanol and aqueous extracts obtained from two endemic plants, *Dianthus elegans* var. *actinopetalus* (Fenzl) Reeve and *Linum ciliatum* Hayek, were investigated for their phenolic composition, antioxidant activities, cytotoxic potential, and supported by in silico molecular docking analysis. High-performance liquid chromatography revealed distinct phenolic acid and flavonoid profiles among the extracts, with the methanol extract of *L. ciliatum* (LME) exhibiting the richest phenolic content. Consistently, LME showed the highest total phenol (314.69 µg/mg) and total flavonoid (91.61 µg/mg) levels. Antioxidant capacity was evaluated using DPPH radical scavenging and metal chelation assays, in which LME demonstrated the strongest activity with the lowest IC₅₀ values (45.49 and 40.10 µg/mL, respectively), highlighting the influence of extraction solvent on bioactivity. Cytotoxic effects were assessed on Mahlavu and MCF-7 cell lines at 24, 48, and 72 h. Both methanol and aqueous extracts of *L. ciliatum* exhibited time-dependent cytotoxicity, with LME showing a remarkably low IC₅₀ value of 0.66 µg/mL on MCF-7 cells after 72 h. To gain insight into the molecular basis of the observed cytotoxic effects, *in silico* molecular docking was performed using the anti-apoptotic Bcl-2 protein as a target. The major phenolic compounds, particularly chlorogenic acid, quercetin, and naringenin, exhibited strong binding affinities toward Bcl-2, suggesting a possible involvement of apoptosis-related pathways. In contrast, none of the extracts showed antibacterial activity against the tested Gram-positive or Gram-negative bacteria. Overall, the findings indicate that methanol extraction is more effective for obtaining phenolic-rich extracts with pronounced antioxidant and anticancer potential.

## Introduction

In the human body, cells continuously undergo metabolic reactions to generate energy, during which oxygen-containing molecules known as free radicals are produced as natural byproducts. Under physiological conditions, the presence of these molecules at controlled levels is considered normal. However, an imbalance between free radical production and the body’s antioxidant defense mechanisms leads to oxidative stress. Oxidative stress is a pathological condition characterized by the detrimental effects of excessive free radicals, which may result in various adverse biological outcomes (Chaudhary et al. 2023a; Jomova et al. 2023). The primary cause of oxidative stress is either the excessive generation of free radicals or the body’s impaired capacity to effectively neutralize and eliminate them. Various exogenous and endogenous factors contribute to increased free radical production, including environmental exposures such as cigarette smoking, air pollution, and excessive sunlight; dietary factors such as imbalanced nutrition and insufficient antioxidant intake; the use of certain medications and exposure to toxins; chronic inflammatory conditions; and medical radiation therapies (Milesi 2006; Wronka et al. 2022). Oxidative stress, which can cause many adverse effects in the body, may lead to various health problems. Free radicals can damage cellular components such as deoxyribonucleic acid (DNA), lipids, and proteins. This damage can disrupt the normal functioning of cells and may ultimately lead to cell death (Das 2023). Oxidative stress, which can accelerate cellular aging and enhance the signs of aging, increases the risk of chronic diseases such as heart disease, diabetes, cancer, and neurodegenerative disorders (Rana and Gautam 2022). In addition, this process, which can weaken the immune system and increase vulnerability to infections, triggers inflammatory responses and leads to chronic inflammation (Zhai et al. 2024).

2,2-Diphenyl-1-picrylhydrazyl (DPPH) is a stable free radical compound widely used as an indicator of oxidative stress, particularly in the assessment of antioxidant activity. Its relevance to oxidative stress stems from its ability to model free radical behavior in experimental systems. While DPPH serves as a representative free radical associated with oxidative stress, antioxidants are compounds that counteract oxidative damage by neutralizing free radicals and thereby mitigating the harmful effects of oxidative stress (Chen et al. 2023b). Antioxidants work to balance oxidative stress by neutralizing free radicals and preventing oxidative damage. Therefore, DPPH radical scavenging assays are used to evaluate the capacity of antioxidants to reduce oxidative stress (Bendeif et al. 2023; Mota et al. 2023). Antioxidants neutralize DPPH by converting it into colorless DPPH-H. DPPH radical scavenging assays help demonstrate that antioxidants may provide protection against health problems caused by oxidative stress (Ngueumaleu et al. 2023). Hence, DPPH radical scavenging assays are an important research tool for assessing the ability of antioxidants to reduce oxidative stress and for understanding health issues associated with oxidative stress (Gerasimova et al. 2022).

The relationship between metal ions and oxidative stress is highly significant. There is evidence suggesting that metal ions may be one of the main causes of oxidative stress and are involved in the underlying mechanisms of many health problems (Han 2023; Roy et al. 2023; Xu et al. 2023). Certain metal ions, particularly transition metals such as iron (Fe) and copper (Cu), can participate in redox reactions that promote the generation of free radicals, thereby contributing to oxidative stress. These reactive species can damage biomolecules, including oxygen-containing compounds. For instance, ferrous iron (Fe²⁺) reacts with hydrogen peroxide (H₂O₂) to produce highly reactive hydroxyl radicals (•OH). Such processes occur through well-established mechanisms, including the Fenton and Haber–Weiss reactions, in which metal ions and hydrogen peroxide collectively drive the formation of potent reactive oxygen species, notably hydroxyl radicals (Huang et al. 2004; Brudzynski 2023).

Some metal ions may inhibit the activity of antioxidant enzymes or interact with antioxidants, thereby enhancing the effects of oxidative stress. For instance, copper ions can inhibit the enzyme superoxide dismutase (SOD), weakening the cellular antioxidant defense system (Jomova et al. 2022). Another effect of metal ions associated with oxidative stress is the initiation of a reaction called lipid peroxidation of cellular membranes. This process involves the oxidative damage of the lipid components of cell membranes by free radicals, leading to membrane injury (Korotkov 2023; Zhao et al. 2023a). The impact of metal ions on oxidative stress can contribute to the development and progression of chronic diseases. For example, metals such as copper and iron have been linked to neurodegenerative diseases like Alzheimer’s and Parkinson’s disease (Acevedo et al. 2019; Mateo et al. 2023). Moreover, metal ions can interfere with antioxidant activity or directly interact with antioxidant compounds, thereby limiting their capacity to neutralize reactive species. This impairment of antioxidant defenses can further exacerbate oxidative stress. Overall, the interplay between metal ions and oxidative stress highlights the role of metal ions in promoting oxidative damage through multiple mechanisms, including enhanced free radical generation, suppression of antioxidant defense systems, and disruption of cellular membrane integrity. Consequently, maintaining tight regulation of metal ion homeostasis and ensuring an appropriate balance between metal ions and antioxidants are critical for mitigating the deleterious effects of oxidative stress (Martín Giménez et al. 2021; Kador and Salvi 2022). Metal chelation is an important strategy for controlling and reducing oxidative stress. This process works by binding free metal ions in the body (such as iron or copper), thereby preventing these metals from participating in oxidative reactions and promoting free radical production. As a result, it helps protect cellular components and prevent oxidative damage. The metal chelation process is carried out by a compound known as a chelating agent. Chelating agents bind to metal ions, preventing them from taking part in oxidative reactions. There are many natural and synthetic chelating agents (Bellotti and Remelli 2021; Gulcin and Alwasel 2022). For example, ethylenediaminetetraacetic acid (EDTA) is widely used in medical and industrial applications due to its strong metal-binding capacity. In addition, some natural compounds also exhibit metal-chelating properties. For instance, phytic acid—a compound found in plants—has the ability to bind metal ions and therefore shows potential as a metal chelator (Singh and Prasad 2020). Metal chelation can also be applied in the treatment of certain medical conditions. For example, in patients with excessive iron accumulation, iron chelating agents can be used to remove excess iron from the body, preventing iron-induced oxidative damage. In summary, metal chelation is an effective strategy for controlling and mitigating oxidative stress. By preventing free metal ions from participating in oxidative reactions, it reduces free radical production and minimizes the harmful effects of oxidative stress. Therefore, metal chelation plays an important role in health and medical applications (Kontoghiorghes 2022; Ravalli et al. 2022).

The relationship between oxidative stress and cancer is a topic that has attracted significant interest from researchers worldwide. It is well known that oxidative stress can lead to DNA damage caused by free radicals within cells. This DNA damage may enable cells to acquire the ability to grow and divide abnormally, which can trigger the onset of cancer (Wang et al. 2023; Zhang et al. 2024). Chronic inflammation, which can promote cancer development and support the growth of cancer cells, may also result from oxidative stress. Oxidative stress can cause lipid peroxidation of cellular membranes, disrupting their structural integrity and preventing cells from performing their normal functions (Long et al. 2021; Rochette et al. 2023). Although therapies such as radiotherapy and chemotherapy are designed to target cancer cells, they can also harm normal cells and increase oxidative stress levels. In conclusion, given that oxidative stress can contribute to the initiation and progression of cancer, research has focused on controlling and balancing this process. The findings from such studies may provide valuable strategies to reduce cancer risk and improve therapeutic outcomes (Guo et al. 2023; Liao and Meng 2023).

Considering studies showing that certain antioxidant compounds can inhibit the growth and reproduction of microorganisms, it is known that these compounds—developed by plants as part of their natural defense mechanisms—protect plants from microbes and pathogens (Shala and Gururani 2021). Some antioxidants can exert antimicrobial effects by affecting the cell membranes of microorganisms or disrupting their metabolic processes. For example, certain polyphenols have been shown to inhibit the growth of bacteria and fungi (El Moussaoui et al. 2019). Antioxidants are also used for food preservation purposes. By adding antioxidants to food products, the risks of oxidative degradation and microbial contamination can be reduced. Therefore, the food industry frequently uses antimicrobial and antioxidant compounds to extend shelf life and maintain food quality. Overall, the relationship between antioxidant and antimicrobial properties demonstrates that antioxidants are valuable compounds with both significant health benefits and practical applications in food preservation (Hong et al. 2023). Plant-derived compounds and antioxidants constitute key bioactive components of many natural plant sources and play a crucial role in the regulation of oxidative stress. These compounds possess the capacity to scavenge free radicals and attenuate oxidative damage (Bjørklund et al. 2018). Natural antioxidants abundantly present in plant-based foods—including phenolic compounds, flavonoids, carotenoids, and vitamin C—interact with reactive species and thereby contribute to the reduction of oxidative stress. This protective effect helps prevent damage to essential cellular macromolecules such as DNA, lipids, and proteins (Pal and Dubey 2013). In addition, certain plant-derived compounds can modulate inflammatory pathways, suggesting that their anti-inflammatory properties may further contribute to the alleviation of oxidative stress. Collectively, these findings underscore the significant role of plant-derived products in preserving cellular integrity and mitigating oxidative stress (Yong et al. 2020).

Endemic plants are species that are native to a specific geographical region and naturally grow only in that area. The natural compounds found in these plants are of great significance in many ways. These compounds play a critical role in preserving the biodiversity of such species while also providing scientists and botanists with opportunities to discover new plant species and metabolites (Dawra et al. 2022).

Endemic plants are integral to the maintenance of regional ecosystem functionality, as they form a fundamental component of local flora and interact dynamically with other plant species to support ecological stability. Beyond their ecological significance, endemic plants also play an important role in regional agriculture and food systems, where they are frequently incorporated into local food supply chains and contribute to the nutritional needs of local populations. Moreover, the cultivation and conservation of endemic plant species can provide meaningful support to local economies (Singh and Chaturvedi 2017). In addition to these roles, endemic plants represent valuable sources of natural bioactive compounds with relevance to both traditional medicine and modern pharmaceutical research. These compounds may exhibit diverse therapeutic properties and contribute to the discovery and development of novel drugs. Notably, bioactive constituents derived from certain endemic plant species have been reported to be effective in the treatment of cancer, infectious diseases, and other health conditions (Grafakou et al. 2022; Emsen et al. 2023; Otero et al. 2023).

In the present study, the antioxidant, cytotoxic, and antimicrobial activities of the endemic plant species *Dianthus elegans* var. *actinopetalus* (Fenzl) Reeve and *Linum ciliatum* Hayek are, to the best of our knowledge, being investigated for the first time in the literature. However, several studies examining various biological activities of *Dianthus* and *Linum* species collected from different regions have attracted attention (Boukeria et al. 2020; Mkedder et al. 2021; Baqer et al. 2023). Based on previous findings on species belonging to the same genera, we aimed to investigate the biological activities of these endemic plants, which are predicted to be rich in bioactive compounds. The main objective of our project is to evaluate the complementary roles of methanol and aqueous extracts obtained from *D. elegans* var. *actinopetalus* and *L. ciliatum* in addition to current cancer treatment methods applied to cancerous liver (Mahlavu) and breast (MCF-7) cells. Within this scope, the study aims to determine the antioxidant activities of the extracts, examine their cytotoxic effects on cancer cells, reveal their antimicrobial potential against various bacteria, and, considering the obtained data, assess the possible contributions of these plants to cancer therapy or health protection.

## Materials and methods

### Collection and identification of plant samples

This study was carried out on two species distributed in the Karaman province: *D. elegans* var. *actinopetalus* and *L. ciliatum*—one being endemic to Türkiye and the other locally endemic to Karaman. *D. elegans* var. *actinopetalus* ve *L. ciliatum* is a perennial herbaceous plant distributed in the Mediterranean region at altitudes between 1000 and 1800 m. It grows in rocky and cliff areas near villages located in the southeastern part of Karaman, close to the Taurus Mountains. Its flowering period occurs between July and September. *L. ciliatum* species, on the other hand, is found within the boundaries of Kayaönü village in the Ayrancı district of Karaman province. This species flowers in July and prefers slopes, dirt road edges, and footpaths as its natural habitat (Tugay et al. 2007; Güner et al. 2012; Sezer 2012).

### Preparation of dried extracts from plant samples

Plant materials were first subjected to mild heating using a low-temperature evaporator. Subsequently, all samples were ground into fine powder with the aid of liquid nitrogen and a mortar. Approximately 10 g of the powdered material was extracted separately with water and methanol solvents employing a Soxhlet apparatus for 48 h. The resulting extracts were filtered through Whatman No. 1 paper, and the solvents were removed using a rotary evaporator maintained at 40 °C. The concentrated extracts were then freeze-dried to obtain completely dried powders. The dried residues were stored in sterile containers at 4 °C until further analysis. The percentage of extraction yield was determined using the formula below (Ulusu et al. 2025).

$$\:\mathrm{E}\mathrm{x}\mathrm{t}\mathrm{r}\mathrm{a}\mathrm{c}\mathrm{t}\mathrm{i}\mathrm{o}\mathrm{n}\:\mathrm{y}\mathrm{i}\mathrm{e}\mathrm{l}\mathrm{d}\:\left({\%}\right)=\left[\frac{\mathrm{A}\mathrm{m}\mathrm{o}\mathrm{u}\mathrm{n}\mathrm{t}\:\mathrm{d}\mathrm{r}\mathrm{y}\:\mathrm{e}\mathrm{x}\mathrm{t}\mathrm{r}\mathrm{a}\mathrm{c}\mathrm{t}\:\left(\mathrm{g}\right)}{\mathrm{A}\mathrm{m}\mathrm{o}\mathrm{u}\mathrm{n}\mathrm{t}\:\mathrm{d}\mathrm{r}\mathrm{y}\:\mathrm{p}\mathrm{l}\mathrm{a}\mathrm{n}\mathrm{t}\:\mathrm{s}\mathrm{a}\mathrm{m}\mathrm{p}\mathrm{l}\mathrm{e}\:\left(\mathrm{g}\right)}\right]\times\:100$$

### Analysis of principal phenolic compounds via HPLC system

High-purity solvents including acetonitrile (≥ 99.9%), methanol (≥ 99.8%), acetic acid (≥ 99.8%), and ultrapure water, all of HPLC grade, were utilized in the analysis. Standard reference compounds—chlorogenic acid, cinnamic acid, *p*-coumaric acid, 8 − 4’-dehydrodiferulic acid, 4,2’-dihydroxychalcone 4-glucoside, epicatechin, gallic acid, isoferulic acid, naringenin, phloridzin, protocatechuic acid, quercetin, resveratrol, and rutin—were purchased from Sigma Chemical Co. (USA). Each phenolic standard was dissolved in methanol to prepare a stock solution (500 mg/L) and stored at 4 °C in the absence of light. Working standard solutions were freshly prepared each day through serial dilution with a 1:1 (v/v) mixture of methanol and water.

The chromatographic analysis was conducted using an Agilent 1260 Infinity HPLC system equipped with a column oven (G1316A), autosampler (G1329B), quaternary pump (G1311C), and diode array detector (G1311D) capable of scanning between 190 and 800 nm. Separation was achieved on an ACE 5 C18 column (5 μm, 100 Å, 250 × 4.6 mm). The injection volume was maintained at 20 µL, with the column temperature set at 25 °C and a constant flow rate of 1.0 mL/min.

The mobile phase consisted of three components: phase A (water with 2% acetic acid, v/v), phase B (acetonitrile with 0.5% acetic acid, v/v), and phase C (pure acetonitrile). The gradient elution program was as follows: 0–30 min, 90% A and 10% B; 30–60 min, 80% A and 20% B; 60–62 min, 55% A and 45% B; 62–77 min, 40% B and 60% C; 77–80 min, 90% A and 10% B. After each run, the initial mobile phase composition was maintained for 5 min to allow column equilibration (Ulusu et al. 2025).

### Measurement of antioxidant capacity

#### Quantification of total flavonoid content

The total flavonoid concentration of methanolic and aqueous plant extracts was determined using quercetin as the reference compound. For the assay, 50 µL of each extract (400 µg/mL) and quercetin standard were dispensed into the wells of a 96-well microplate. Subsequently, 215 µL of 80% ethanol, 5 µL of 10% aluminum nitrate, and 5 µL of 1 M potassium acetate were sequentially added. The reaction mixtures were incubated at ambient temperature for 40 min. After incubation, absorbance readings were recorded at 415 nm using a microplate reader. The total flavonoid content of each sample was expressed as quercetin equivalents (QE) derived from a calibration curve prepared with standard quercetin solutions (Ulusu et al. 2025).

#### Quantification of total phenolic compounds

The total phenolic concentration of methanol and water extracts was determined using the Folin–Ciocalteu colorimetric method, with gallic acid serving as the calibration standard. Aliquots of 20 µL from each extract (400 µg/mL) and gallic acid standard were transferred into individual wells of a 96-well microplate. Subsequently, 20 µL of 2 N Folin–Ciocalteu reagent was added to each well, and the plate was mixed gently and incubated in the dark for 3 min. This step was followed by the addition of 20 µL of 35% (w/v) sodium carbonate solution and 140 µL of distilled water. The mixtures were again incubated for 10 min in darkness. Absorbance readings were then recorded at 725 nm using a microplate reader. Total phenolic content was expressed as gallic acid equivalents (GAE), calculated from a standard calibration curve prepared with gallic acid (Ulusu et al. 2025).

### Evaluation of free radical scavenging capacity

The ability of methanolic and aqueous plant extracts to scavenge DPPH radicals was determined using a modified version of the standard DPPH assay. Serial dilutions of each extract were prepared to obtain final concentrations of 12.5, 25, 50, 100, 200, and 400 µg/mL in the wells of a microplate. Subsequently, 20 µL of each extract was combined with 180 µL of 0.06 mM DPPH solution prepared in methanol. The mixtures were incubated in darkness for 60 min to allow the reaction to proceed. After incubation, absorbance values were recorded at 517 nm using a microplate reader. The DPPH radical scavenging activity of the samples was expressed as a percentage inhibition, calculated using the corresponding formula (Kok et al. 2023).

$$\:\mathrm{R}\mathrm{a}\mathrm{d}\mathrm{i}\mathrm{c}\mathrm{a}\mathrm{l}\:\mathrm{s}\mathrm{c}\mathrm{a}\mathrm{v}\mathrm{e}\mathrm{n}\mathrm{g}\mathrm{i}\mathrm{n}\mathrm{g}\:\mathrm{a}\mathrm{c}\mathrm{t}\mathrm{i}\mathrm{v}\mathrm{i}\mathrm{t}\mathrm{y}\:\left({\%}\right)=\left[\frac{\mathrm{C}\mathrm{o}\mathrm{n}\mathrm{t}\mathrm{r}\mathrm{o}\mathrm{l}\:\mathrm{a}\mathrm{b}\mathrm{s}\mathrm{o}\mathrm{r}\mathrm{b}\mathrm{a}\mathrm{n}\mathrm{c}\mathrm{e}\:-\:\mathrm{E}\mathrm{x}\mathrm{t}\mathrm{r}\mathrm{a}\mathrm{c}\mathrm{t}\:\mathrm{a}\mathrm{b}\mathrm{s}\mathrm{o}\mathrm{r}\mathrm{b}\mathrm{a}\mathrm{n}\mathrm{c}\mathrm{e}}{\mathrm{C}\mathrm{o}\mathrm{n}\mathrm{t}\mathrm{r}\mathrm{o}\mathrm{l}\:\mathrm{a}\mathrm{b}\mathrm{s}\mathrm{o}\mathrm{r}\mathrm{b}\mathrm{a}\mathrm{n}\mathrm{c}\mathrm{e}}\right]\times\:100$$

## Assessment of metal ion chelating capacity

The metal ion chelating capacity of methanolic and aqueous plant extracts was analyzed using various concentrations (12.5, 25, 50, 100, 200, and 400 µg/mL) prepared in a 96-well microplate. For each well, 50 µL of extract was mixed with 10 µL of 5 mM ferrozine, 5 µL of 2 mM FeCl₂, and 185 µL of methanol. The mixtures were gently homogenized and left to react at room temperature for 10 min. Following incubation, absorbance readings were recorded at 562 nm using a spectrophotometer. The chelating efficiency of the extracts was expressed as a percentage inhibition, calculated according to the appropriate formula (Kok et al. 2023).

$$\:\mathrm{M}\mathrm{e}\mathrm{t}\mathrm{a}\mathrm{l}\:\mathrm{c}\mathrm{h}\mathrm{e}\mathrm{l}\mathrm{a}\mathrm{t}\mathrm{i}\mathrm{n}\mathrm{g}\:\mathrm{a}\mathrm{c}\mathrm{t}\mathrm{i}\mathrm{v}\mathrm{i}\mathrm{t}\mathrm{y}\:\left({\%}\right)=\left[\frac{\mathrm{C}\mathrm{o}\mathrm{n}\mathrm{t}\mathrm{r}\mathrm{o}\mathrm{l}\:\mathrm{a}\mathrm{b}\mathrm{s}\mathrm{o}\mathrm{r}\mathrm{b}\mathrm{a}\mathrm{n}\mathrm{c}\mathrm{e}\:-\:\mathrm{E}\mathrm{x}\mathrm{t}\mathrm{r}\mathrm{a}\mathrm{c}\mathrm{t}\:\mathrm{a}\mathrm{b}\mathrm{s}\mathrm{o}\mathrm{r}\mathrm{b}\mathrm{a}\mathrm{n}\mathrm{c}\mathrm{e}}{\mathrm{C}\mathrm{o}\mathrm{n}\mathrm{t}\mathrm{r}\mathrm{o}\mathrm{l}\:\mathrm{a}\mathrm{b}\mathrm{s}\mathrm{o}\mathrm{r}\mathrm{b}\mathrm{a}\mathrm{n}\mathrm{c}\mathrm{e}}\right]\times\:100$$

### Determination of cytotoxic activity

#### Cell culture

Cancerous liver (Mahlavu) and breast (MCF-7) cells were cultured in Dulbecco’s Modified Eagle Medium (DMEM) supplemented with high glucose, L-glutamine, sodium pyruvate, 10% fetal bovine serum (FBS), and 1% antibiotic (Penicillin-Streptomycin). During the culturing process, a 5% CO₂ and 95% humidity incubator (Sanyo MCO 17AIC, USA) was used. When the cells reached the desired confluence (90%), they were detached using trypsin/EDTA and washed with fresh medium. The cells were then transferred back into growth media, stained with trypan blue, and counted using a cell counter (TC-10, Bio-RAD, Germany).

#### 3-(4,5-Dimethylthiazol-2-yl)-2,5-diphenyltetrazolium bromide (MTT) assay

For the present study, the required concentrations (1.5625, 3.125, 6.25, 12.5, 25, 50, 100, 200, and 400 µg/mL) were determined based on the calculated IC₅₀ values. As a negative control group, the relevant cell culture medium containing no more than 0.5% dimethyl sulfoxide (DMSO) as a solvent was used. Mitomycin-C (C₁₅H₁₈N₄O₅, Sigma, St. Louis/MO, USA) was employed as a positive control. The IC₅₀ value of mitomycin-C on cells was taken as a reference, and its concentrations (0.5, 1, 2, 5, 10, 20, 50, 100, and 200 µM) were accordingly determined.

To evaluate the effects of methanol and aqueous extracts of the plants on the viability of Mahlavu and MCF-7 cells, an MTT cytotoxicity assay was performed. In this assay, MTT, due to its net positive charge and plasma membrane potential, penetrates the cell and is reduced by intracellular NAD(P)H oxidoreductases to purple-colored formazan (Berridge et al. 2005). According to the method, cells were seeded at a density of 1 × 10⁴ cells/well into flat-bottomed 96-well microplates and incubated at 37 °C for 24 h. After 24 h, the medium was replaced with fresh medium containing different concentrations of the plant extracts. The cells were then incubated at 37 °C in a CO₂ incubator for 24, 48, and 72 h. At the end of each incubation period, the plates were removed from the incubator, and 10 µL of MTT reagent was added to each well containing 100 µL of medium and cells. The plate was incubated at 37 °C in a CO₂ incubator for 4 h. After incubation, the medium was carefully removed using a micropipette to preserve the monolayer of cells and the formazan crystals at the bottom of each well. Subsequently, 100 µL of crystal dissolving solution was added to each well, and spectrophotometric readings were taken at 570 nm. Cell viability was calculated using the following formula:

$${Cell\:viability\:(\%)\:=\:}\frac{{Sample\:absorbance}}{{Control\:absorbance}}\times\:100$$

### Antimicrobial analyses

#### Disk diffusion analysis

The disk diffusion method was used to test the antimicrobial effects of the plant extracts against *Staphylococcus aureus* Rosenbach (ATCC 9144), *Bacillus cereus* Frankland & Frankland (NRRL B-3711), *Pseudomonas aeruginosa* (Schroeter) Migula (ATCC 27853), and *Escherichia coli* (Migula) Castellani and Chalmers (ATCC 700728) bacterial strains (Bauer et al. 1966; Matuschek et al. 2014). For this purpose, colonies from the test bacteria grown on nutrient agar solid medium were transferred into Mueller Hinton Broth and incubated at 37 °C with shaking at 180 rpm for 16 h. The density of the bacterial cultures was then adjusted to approximately 1 × 10⁸ CFU/mL (0.5 McFarland Standard) using sterile physiological saline solution (0.85% NaCl). From these adjusted pure culture suspensions, 100 µL was used to inoculate Mueller Hinton agar plates. Subsequently, 6 mm diameter disks were impregnated with 20 µL of plant extract at a concentration of 40 mg/mL and placed on the surface of the inoculated agar plates. The plates were incubated at 37 °C for approximately 16 h. After incubation, the inhibition zones around the disks were measured and evaluated. During the experiments, Gentamicin (10 µg), Tetracycline (30 µg), and Penicillin (10 U) disks were used as standard antibiotics, while disks impregnated only with solvent (water and methanol) served as negative controls.

#### Minimum inhibitory concentration (MIC) analysis

The protocol described by (Wiegand et al. 2008) was applied to determine the minimum concentration of plant extracts that inhibited the growth of *S. aureus*, *B. cereus*, *P. aeruginosa*, and *E. coli* bacterial strains. Briefly, pure bacterial cultures grown in Mueller Hinton Broth medium were adjusted to a density of approximately ~ 1 × 10⁸ CFU/mL (0.5 McFarland Standard) and diluted 1:50 using sterile physiological saline. After dilution, the bacterial suspensions were mixed in equal volumes with plant extract solutions prepared at different concentrations ranging from 0 to 40 mg/mL in 96-well microplates, and the plates were incubated at 37 °C for approximately 16 h. At the end of the incubation period, the plates were visually examined, and the lowest extract concentration that inhibited bacterial growth was recorded as the MIC value. In addition, standard antibiotic solutions prepared at concentrations between 0 and 500 µg/mL were tested under the same conditions, and the MIC values of these antibiotics for the test bacteria were also determined.

#### Molecular Docking analysis

Molecular docking studies were conducted to investigate the potential interactions between major phenolic compounds identified in the plant extracts and the anti-apoptotic Bcl-2 protein. The three-dimensional crystal structure of human Bcl-2 was retrieved from the Protein Data Bank (PDB ID: 6O0K). Prior to docking simulations, the protein structure was prepared using ChimeraX version 1.11. Preparation steps included the removal of water molecules and co-crystallized ligands, followed by the addition of polar hydrogen atoms and the assignment of appropriate atomic charges to ensure structural optimization for docking analysis.

Ligand structures, including chlorogenic acid, quercetin, naringenin, 8–4′-dehydrodiferulic acid, gallic acid, and resveratrol, were obtained from the PubChem database in SDF format. All ligand molecules were subjected to energy minimization to achieve stable conformations prior to docking.

Molecular docking simulations were performed using PyRx software, which incorporates AutoDock Vina as the docking engine. Protein and ligand structures were converted into PDBQT format within PyRx. The docking grid box was defined to encompass the active binding pocket of Bcl-2 and was centered based on the position of the co-crystallized ligand present in the crystal structure. Docking calculations were carried out using default AutoDock Vina parameters, and multiple binding conformations were generated for each ligand.

The best-ranked docking poses were selected based on the lowest binding affinity values (kcal/mol) and acceptable root mean square deviation (RMSD) values. The resulting docked complexes were further analyzed to evaluate ligand–protein interactions, binding orientations, and interaction types.

Visualization and detailed analysis of molecular interactions, including hydrogen bonds, van der Waals interactions, and aromatic interactions, were performed using Discovery Studio Visualizer version 25. Two-dimensional (2D) interaction diagrams and three-dimensional (3D) binding mode representations were generated to identify key amino acid residues involved in ligand binding and to elucidate the interaction patterns between Bcl-2 and the selected phenolic compounds.

### Data processing and statistical evaluation

The biological activity data obtained from the plant extracts were statistically analyzed using one-way analysis of variance (ANOVA), followed by Duncan’s multiple range test for mean separation. Median inhibitory concentration (IC₅₀) values were estimated through probit regression analysis. To explore the interrelationships among DPPH radical scavenging, metal chelating, and cytotoxic activities, three-dimensional (3D) density plots were generated. All statistical computations were performed using SPSS software (version 27.0; IBM Corp., Armonk, NY, USA). Furthermore, to visualize the correlations and clustering patterns between antioxidant assays (DPPH and metal chelation), cytotoxic activities, and HPLC-derived phenolic profiles, heatmap and hierarchical clustering analyses were conducted. These analyses were based on Ward’s minimum variance method using R software (version 4.1.0) within the RStudio interface (version 1.4.1717).

## Results

### Extraction yield

**Fig. 1:**
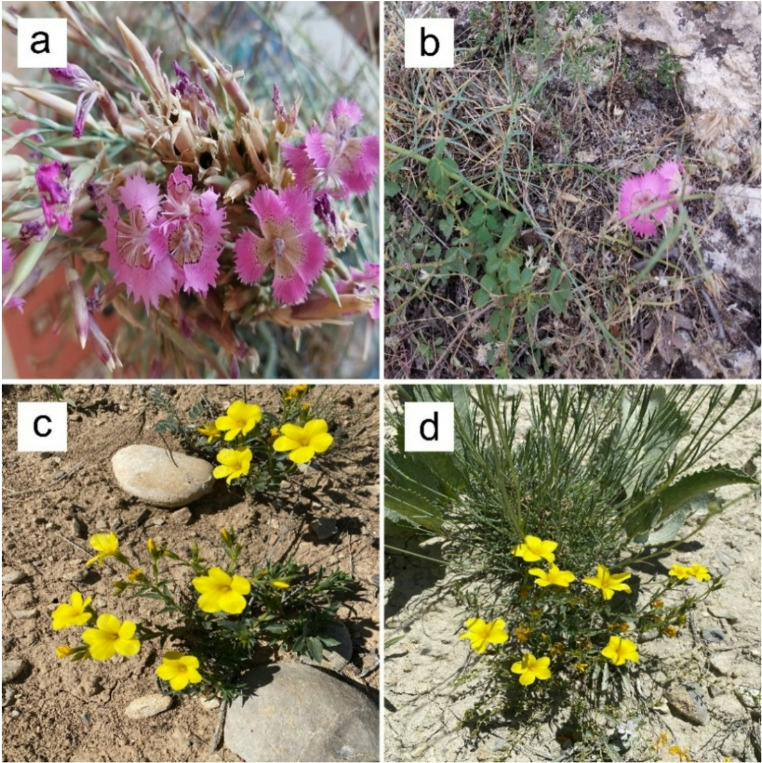
Images of **a**,** b*** D. elegans* var. *actinopetalus.* **c**,** d ***L. ciliatum* in their natural habitat

The extraction yields of the extracts obtained from *D. elegans* var. *actinopetalus* (Fig. 1a, b) and *L. ciliatum* (Fig. 1c, d) (DME: Methanol extract of *D. elegans* var. *actinopetalus*; DWE: Water extract of *D. elegans* var. *actinopetalus*; LME: Methanol extract of *L. ciliatum*; LWE: Water extract of *L. ciliatum*) are presented in Table 1. The extraction yield of *D. elegans var. actinopetalus* varied depending on the solvent used. The methanol extract showed a higher yield of 10.66%, while the aqueous extract yielded 5.06%. In contrast, *L. ciliatum* exhibited the opposite trend, with the aqueous extract providing a higher yield of 13.86% compared to 9.73% for the methanol extract. These results indicate that extraction yield depends on both the plant species and the type of solvent used.

**Table 1:** Yield (%) of obtained the plant extracts

Extract	Yield (w/w)
Methanol extract of *D. elegans* var. *actinopetalus*	10.66
Water extract of *D. elegans* var. *actinopetalus*	5.06
Methanol extract of *L. ciliatum*	9.73
Water extract of *L. ciliatum*	13.86

### Identification and quantification of main phenolic compounds by HPLC

**Fig. 2:**
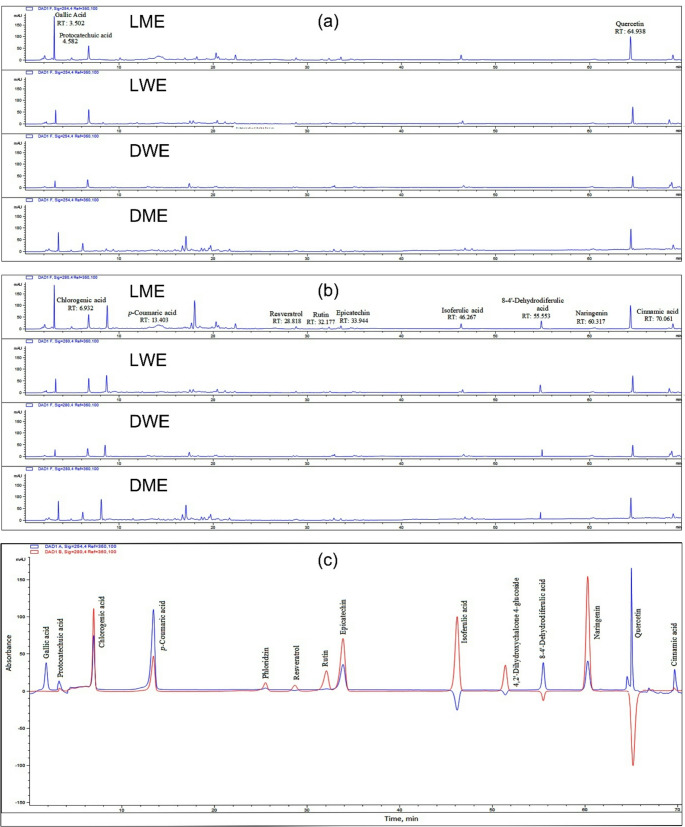
HPLC chromatograms of the extracts recorded at **a** 254 nm and **b** 280 nm, along with **c** chromatograms of the standard compounds measured at both 254 and 280 nm. DME: Methanol extract of *D. elegans* var. *actinopetalus*; DWE: Water extract of *D. elegans* var. *actinopetalus*; LME: Methanol extract of *L. ciliatum*; LWE: Water extract of *L. ciliatum*

The phenolic acid and flavonoid contents of the methanol and water extracts obtained from the plants were analyzed using the HPLC method, and their chromatograms are presented in Fig. 2. These chromatograms were evaluated comparatively with reference standards for the identification and quantification of each compound, and based on the obtained data, the phenolic and flavonoid profiles of the extracts were determined. The quantitative data obtained from the analyses are presented in detail in Tables 2 and 3.

**Table 2:** Quantities (µg/g) of some phenolic acids in the plant extracts

Phenolic acids	Detection wavelength (nm)	RT (min)	DME	DWE	LME	LWE
Chlorogenic acid	280	6.932	421.1 ± 4.04	328.3 ± 3.76	523.83 ± 3.61	481.1 ± 2.18
Cinnamic acid	280	70.061	4.704 ± 0.32	4.182 ± 0.14	6.883 ± 0.28	6.595 ± 0.37
*p*-Coumaric acid	280	13.403	3.066 ± 0.06	2.215 ± 0.12	4.592 ± 0.16	3.206 ± 0.12
8 − 4’-Dehydrodiferulic acid	254	55.553	10.23 ± 1.12	7.486 ± 0.71	17.07 ± 0.78	15.43 ± 0.45
Gallic Acid	254	3.502	552.26 ± 6.99	412.87 ± 4.57	564.42 ± 12.2	494.0 ± 11.06
Isoferulic acid	280	46.267	2.534 ± 0.05	2.148 ± 0.06	3.134 ± 0.01	2.771 ± 0.04
Protocatechuic acid	254	4.582	0.346 ± 0.01	nd	0.145 ± 0.01	nd

Each value is expressed as mean ± standard deviation (*n* = 3). nd: not determined. DME: Methanol extract of *D. elegans* var. *actinopetalus*; DWE: Water extract of *D. elegans* var. *actinopetalus*; LME: Methanol extract of *L. ciliatum*; LWE: Water extract of *L. ciliatum*

**Table 3:** Quantities (µg/g) of some some flavonoids in the plant extracts

Flavonoids	Detection wavelength (nm)	RT (min)	DME	DWE	LME	LWE
4,2’-Dihydroxychalcone 4-glucoside	280	51.456	nd	nd	nd	nd
Epicatechin	280	33.944	1.825 ± 0.12	nd	2.137 ± 0.07	nd
Naringenin	280	60.317	1.295 ± 0.13	1.311 ± 0.13	1.574 ± 0.04	1.427 ± 0.07
Phloridzin	280	25.587	nd	nd	nd	nd
Quercetin	254	64.938	66.66 ± 2.18	48.33 ± 2.40	74.40 ± 2.93	58.81 ± 5.78
Resveratrol	280	28.818	0.831 ± 0.05	0.322 ± 0.08	1.239 ± 0.13	0.934 ± 0.02
Rutin	280	32.177	0.241 ± 0.04	0.226 ± 0.02	0.323 ± 0.02	0.321 ± 0.01

Each value is expressed as mean ± standard deviation (*n* = 3). nd: not determined. DME: Methanol extract of *D. elegans* var. *actinopetalus*; DWE: Water extract of *D. elegans* var. *actinopetalus*; LME: Methanol extract of *L. ciliatum*; LWE: Water extract of *L. ciliatum*

Table 2 shows the amounts of the main phenolic acids in the plant extracts. Chlorogenic acid was ranked as LME > LWE > DME > DWE, with the highest value found in LME (523.83 ± 3.61 µg/g). In terms of gallic acid, the order was observed as LME (564.42 ± 12.2 µg/g) > DME (552.26 ± 6.99 µg/g) > LWE (494.0 ± 11.06 µg/g) > DWE (412.87 ± 4.57 µg/g). Cinnamic acid and p-coumaric acid were ordered as LME > LWE > DME > DWE, with cinnamic acid (6.883 ± 0.28 µg/g) and p-coumaric acid (4.592 ± 0.16 µg/g) determined in LME. 8 − 4’-Dehydrodiferulic acid was ranked as LME (17.07 ± 0.78 µg/g) > LWE (15.43 ± 0.45 µg/g) > DME (10.23 ± 1.12 µg/g) > DWE (7.486 ± 0.71 µg/g). The amount of isovanillic acid was also found to follow the order LME > LWE > DME > DWE.

Table 3 presents the higher quantities of the main flavonoids in the plant extracts. In terms of quercetin, the highest concentration was observed as LME (74.40 ± 2.93 µg/g) > DME (66.66 ± 2.18 µg/g) > LWE (58.81 ± 5.78 µg/g) > DWE (48.33 ± 2.40 µg/g). Resveratrol was found in the order of LME (1.239 ± 0.13 µg/g) > LWE (0.934 ± 0.02 µg/g) > DME (0.831 ± 0.05 µg/g) > DWE (0.322 ± 0.08 µg/g). Epicatechin followed the order LME (2.137 ± 0.07 µg/g) > DME (1.825 ± 0.12 µg/g), while naringenin was determined as LME (1.574 ± 0.04 µg/g) > LWE (1.427 ± 0.07 µg/g) > DWE (1.311 ± 0.13 µg/g) > DME (1.295 ± 0.13 µg/g). Rutin followed the order LME (0.323 ± 0.02 µg/g) > LWE (0.321 ± 0.01 µg/g) > DME (0.241 ± 0.04 µg/g) > DWE (0.226 ± 0.02 µg/g).

In general evaluation, the highest quantities of both phenolic acids and flavonoids were detected in the LME extract, followed by LWE, DME, and DWE extracts, respectively. These findings indicate that methanol is a more effective solvent for the extraction of phenolic compounds; therefore, methanol extracts may possess higher values in terms of biological activity potential.

### Determination of antioxidant compounds

The total phenolic and total flavonoid contents of the plant extracts were examined. In terms of total phenolic content, LME showed the highest value with 314.69 ± 8.36 µg/mg, followed by LWE (277.79 ± 8.44 µg/mg), DME (245.76 ± 4.41 µg/mg), and DWE (199.16 ± 7.08 µg/mg). This ranking indicates that methanol extracts, particularly LME, are richer in phenolic compounds. A similar trend was also observed for total flavonoid contents. LME exhibited the highest flavonoid amount with 91.61 ± 1.25 µg/mg, while LWE (74.18 ± 1.12 µg/mg), DME (70.58 ± 0.52 µg/mg), and DWE (63.64 ± 1.79 µg/mg) extracts showed lower values. These results demonstrate that both phenolic and flavonoid contents vary depending on the extraction method and solvent type. The superior performance of LME in terms of both total phenolic and flavonoid contents indicates that LME possesses strong antioxidant potential. On the other hand, the lower phenolic and flavonoid contents of the water extracts compared to the methanol extracts suggest that water has limited capacity to dissolve certain phenolic and flavonoid compounds. Overall, the obtained data reveal that methanol extraction is more effective for efficiently obtaining antioxidant compounds (Table 4).

**Table 4:** Antioxidant compounds of the plant extracts (µg/mg)

Treatment	Total phenol (Gallic acid equivalent)	Total flavonoid (Quercetin equivalent)
DME	245.76 ± 4.41 c	70.58 ± 0.52 c
DWE	199.16 ± 7.08 d	63.64 ± 1.79 d
LME	314.69 ± 8.36 a	91.61 ± 1.25 a
LWE	277.79 ± 8.44 b	74.18 ± 1.12 b

Each value is expressed as mean ± standard deviation (*n* = 5). Values indicated by different letters in the same column differ from each other at the level of *p* < 0.05. DME: Methanol extract of *D. elegans* var. *actinopetalus*; DWE: Water extract of *D. elegans* var. *actinopetalus*; LME: Methanol extract of *L. ciliatum*; LWE: Water extract of *L. ciliatum*

### DPPH radical scavenging activity

**Fig. 3:**
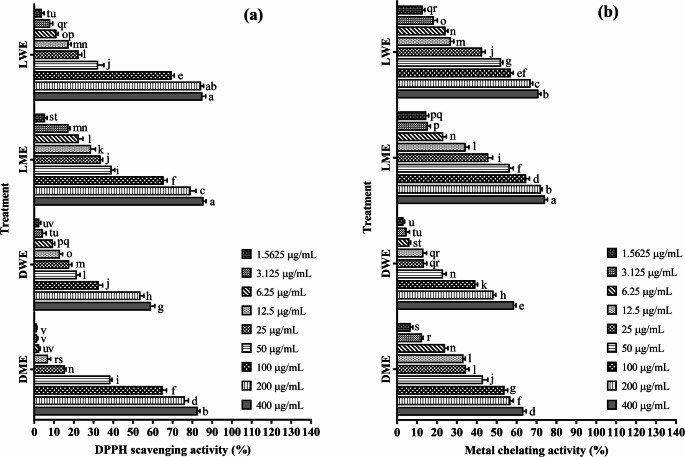
**a** DPPH radical scavenging and **b** metal chelating activities of different extracts from the plants (mean ± standard deviation, *n* = 5). Values indicated by different letters differ from each other at the level of *p* < 0.05. DME: Methanol extract of *D. elegans* var. *actinopetalus*; DWE: Water extract of *D. elegans* var. *actinopetalus*; LME: Methanol extract of *L. ciliatum*; LWE: Water extract of *L. ciliatum*

According to the findings obtained in this study, the DPPH radical scavenging activities of all extracts increased significantly with increasing concentration. In the DPPH assay, particularly the LWE and LME extracts exhibited more than 85% radical scavenging capacity at a concentration of 400 µg/mL, reaching the highest activity levels. In contrast, the DME and DWE extracts showed limited activity at lower concentrations; however, a marked increase was observed as the concentration increased. According to Duncan’s multiple range test results (*p* < 0.05), statistically significant differences were found between groups indicated by different letters in the same graph. For example, LME and LWE were classified in group “a” at higher concentrations, indicating the highest activity levels, whereas DME and DWE were categorized into lower groups such as “r, s, t, u” at lower concentrations. These results indicate that the antioxidant activities of the extracts vary depending on both the extract type and the applied concentration, and that LME and LWE were statistically and significantly superior to the other extracts (Fig. 3a).

The DPPH radical scavenging activity of the plant extracts was further evaluated using IC₅₀ values. LME exhibited the lowest IC₅₀ value (45.49 µg/mL), indicating the strongest radical scavenging capacity. This was followed by LWE (59.00 µg/mL) > DME (80.01 µg/mL) > DWE (234.84 µg/mL), demonstrating that the efficiency of both methanol and water extracts depends on the plant species and extraction method. Gallic acid, used as the standard compound, provided a reference level with an IC₅₀ value of 15.44 µM. The distinctly higher DPPH radical scavenging activity of LME compared to the other extracts may be attributed to methanol’s superior ability to dissolve phenolic and flavonoid compounds. The lowest activity observed in DWE, with an IC₅₀ value of 234.84 µg/mL, suggests that water may not sufficiently dissolve certain radical scavenging compounds. DME and LWE showed moderate activity, highlighting that both solvent type and plant species significantly influence radical scavenging capacity. Overall, the results obtained from the DPPH assay indicate that LME possesses strong antioxidant potential and that methanol extraction is particularly effective in obtaining plant-derived radical scavenging compounds (Table 5).

**Table 5:** IC₅₀ values (µg/mL for plant extracts and µM for standard compounds) obtained from DPPH radical scavenging and metal chelating assays

Activity	Treatment	IC₅₀ (Limits)	Slope ± Standard error of the mean (Limits)
DPPH scavenging	DME	80.01 (74.63–85.91)	1.62 ± 0.04 (1.54–1.71)
	DWE	234.84 (202.42–276.69)	0.91 ± 0.03 (0.84–0.97)
	LME	45.49 (41.36–50.15)	1.01 ± 0.03 (0.95–1.06)
	LWE	59.00 (54.46–64.05)	1.30 ± 0.03 (1.23–1.36)
	Gallic acid	15.44 (14.12–16.90)	1.10 ± 0.02 (1.04–1.16)
Metal chelating	DME	93.37 (80.56–109.58)	0.69 ± 0.02 (0.64–0.75)
	DWE	240.67 (207.67–283.22)	0.92 ± 0.03 (0.86–0.99)
	LME	40.10 (35.77–45.08)	0.80 ± 0.02 (0.74–0.85)
	LWE	55.47 (48.78–63.49)	0.73 ± 0.02 (0.67–0.78)
	EDTA	41.96 (37.89–46.70)	1.06 ± 0.03 (1.00–1.12)

EDTA: Ethylenediaminetetraacetic acid; DME: Methanol extract of *D. elegans* var. *actinopetalus*; DWE: Water extract of *D. elegans* var. *actinopetalus*; LME: Methanol extract of *L. ciliatum*; LWE: Water extract of *L. ciliatum*

### Metal chelating activity

According to the findings obtained in this study, the metal chelating activities of all extracts increased significantly with increasing concentration. The LME extract exhibited the strongest effect with a chelating capacity of 74.27% at 400 µg/mL, followed by LWE. Although DME and DWE extracts showed low activity at lower concentrations, their chelating capacities increased significantly at higher doses. According to Duncan’s multiple range test results (*p* < 0.05), statistically significant differences were observed between groups indicated by different letters in the same graph. The 400 µg/mL concentrations of LME and LWE showed significantly higher activity than all other treatments (Fig. 3b).

The metal chelating activity of the plant extracts was evaluated based on their IC₅₀ values. LME showed the lowest IC₅₀ value (40.10 µg/mL), indicating the highest metal ion binding capacity. This was followed by LWE (55.47 µg/mL) > DME (93.37 µg/mL) > DWE (240.67 µg/mL). EDTA, used as a standard chelating agent, provided a reference level with an IC₅₀ value of 41.96 µM. The markedly higher metal chelating capacity of LME compared to the other extracts suggests that methanol extraction is more effective in dissolving metal-binding phenolic and flavonoid compounds. DWE, with an IC₅₀ value of 240.67 µg/mL, exhibited the lowest activity, indicating that water extracts may not contain enough certain metal-chelating compounds. DME and LWE demonstrated moderate activity, showing that both solvent type and plant species influence metal chelating capacity. Overall, the results obtained from the metal chelating assay indicate that LME possesses strong metal chelating activity and that methanol extraction is more effective in obtaining such antioxidant compounds (Table 5).

### Cytotoxic activity

**Fig. 4:**
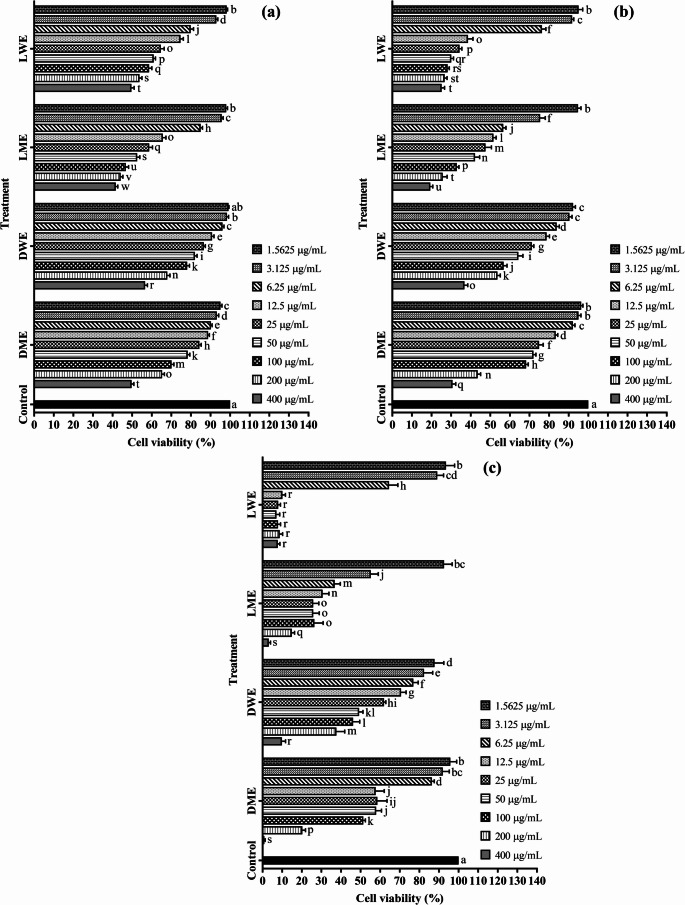
Viability rates of Mahlavu cells treated with plant extracts for **a** 24 h, **b** 48 h, and **c** 72 h (mean ± standard deviation, *n* = 5). Values indicated by different letters differ from each other at the level of *p* < 0.05. DME: Methanol extract of *D. elegans* var. *actinopetalus*; DWE: Water extract of *D. elegans* var. *actinopetalus*; LME: Methanol extract of *L. ciliatum*; LWE: Water extract of *L. ciliatum*

According to the findings obtained in this study, all extracts caused significant differences in cell viability of Mahlavu cells depending on both concentration and exposure time at 24, 48, and 72 h (Fig. 4a–c). Based on Duncan’s multiple range test, the control group was labeled with the letter “a,” while extract-treated groups—especially at higher concentrations—were assigned different letters, indicating statistically significant differences (*p* < 0.05) compared to the control.

Overall, DME and DWE extracts induced a gradual decrease in cell viability with increasing concentrations at all time points, showing the lowest viability rates at 200 and 400 µg/mL. In contrast, LWE and LME extracts exhibited viability rates close to the control at low doses (1.5625–12.5 µg/mL) and in most cases showed relatively higher viability. However, at 72-hour treatments, a significant reduction in viability was also observed for LWE and LME, and these groups shifted to lower statistical subgroups indicating reduced viability.

When the effect of time was examined, viability rates were higher at 24 h, while cytotoxic effects became more pronounced at 48 and especially 72 h. Notably, in the DME treatment group, cell viability dropped below 1% at 72 h. These results indicate that the cytotoxic effects of the extracts were significantly enhanced both by increased dose and prolonged exposure time.

**Fig. 5:**
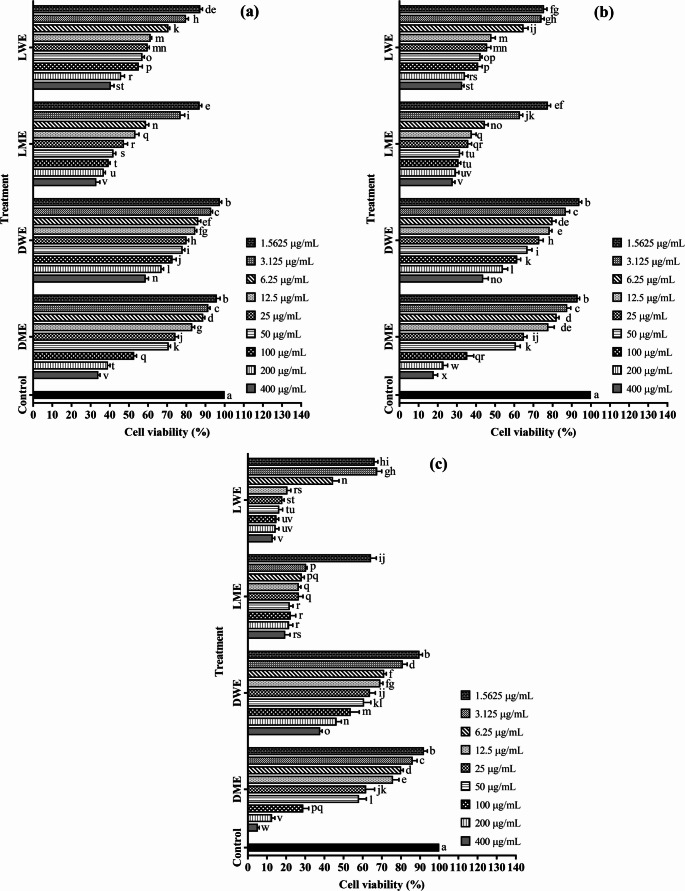
Viability rates of MCF-7 cells treated with plant extracts for **a** 24 h, **b** 48 h, and **c** 72 h (mean ± standard deviation, *n* = 5). Values indicated by different letters differ from each other at the level of *p* < 0.05. DME: Methanol extract of *D. elegans* var. *actinopetalus*; DWE: Water extract of *D. elegans* var. *actinopetalus*; LME: Methanol extract of *L. ciliatum*; LWE: Water extract of *L. ciliatum*

In MCF-7 cells, treatments with different extracts for 24, 48, and 72 h also resulted in statistically significant effects on cell viability depending on both concentration and time (Fig. 5a–c). According to Duncan’s multiple range test, the control group was represented by the letter “a,” whereas extract treatments appeared in distinct letter groups as concentration increased, showing significant (*p* < 0.05) differences from the control.

In general, DME and DWE treatments caused a gradual decrease in cell viability with increasing concentrations, showing the lowest viability rates at 200 and 400 µg/mL, grouped within the “q–w” statistical range. Conversely, LWE and LME treatments exhibited viability levels close to the control at lower concentrations (1.5625–12.5 µg/mL) and were mostly grouped between “b–f.” As exposure time increased, significant decreases in viability were also observed for these extracts, with 72-hour treatments shifting to lower statistical groups such as “p–v.”

Regarding the time factor, cell viability was higher after 24 h, the cytotoxic effect became more pronounced at 48 h, and the most substantial reduction occurred at 72 h. In particular, DME treatments resulted in cell viability dropping below 10% at 72 h, grouping within the lowest statistical categories (“v–w”). These findings reveal that the cytotoxic activity of the extracts in MCF-7 cells strengthened with increasing concentration and longer exposure times, supported by statistically significant differences.

In cytotoxicity tests conducted on both Mahlavu and MCF-7 cell lines, the IC₅₀ values of the plant extracts were evaluated at 24, 48, and 72 h of incubation. In Mahlavu cells after 24 h, LME showed the lowest IC₅₀ value (98.22 µg/mL), exhibiting markedly higher cytotoxicity than DME (587.58 µg/mL) and DWE (652.12 µg/mL). LWE showed moderate activity with an IC₅₀ value of 210.08 µg/mL. Mitomycin-C, used as a standard anticancer compound, provided a reference with an IC₅₀ value of 90.94 µg/mL. A similar trend was observed in MCF-7 cells, where LME demonstrated the strongest cytotoxic activity with an IC₅₀ value of 34.48 µg/mL. LWE and DME exhibited moderate effects with IC₅₀ values of 114.74 µg/mL and 131.22 µg/mL, respectively, while DWE showed the lowest cytotoxicity with an IC₅₀ value of 990.33 µg/mL. Mitomycin-C served as a reference with an IC₅₀ value of 39.61 µM.

When the incubation period was extended to 48 h, a significant decrease in IC₅₀ values was observed for both LME and LWE extracts in Mahlavu and MCF-7 cells, indicating increased cytotoxicity over time. In Mahlavu cells, LME (24.44 µg/mL) and LWE (25.26 µg/mL) showed strong effects; in MCF-7 cells, LME (9.24 µg/mL) and LWE (28.50 µg/mL) were the most effective extracts. Mitomycin-C continued to serve as a reference, with IC₅₀ values of 20.05 µM (Mahlavu) and 9.62 µM (MCF-7).

After 72 h of incubation, cytotoxicity became even more pronounced. In Mahlavu cells, IC₅₀ values ranked as LME (7.01 µg/mL), LWE (8.48 µg/mL), DME (42.71 µg/mL), and DWE (48.52 µg/mL). In MCF-7 cells, LME (0.66 µg/mL), LWE (4.12 µg/mL), DME (36.01 µg/mL), and DWE (122.63 µg/mL) exhibited strong cytotoxic effects. Mitomycin-C was used as a reference with IC₅₀ values of 2.21 µM (Mahlavu) and 0.79 µM (MCF-7).

Overall, the results indicate that LME and LWE extracts possess notably high cytotoxic activity, particularly in MCF-7 cells, whereas DME and DWE extracts show comparatively lower efficacy. Additionally, cytotoxic activity was found to increase with longer incubation times, demonstrating that both extract type and exposure duration are key determinants of cytotoxicity (Table 6).

**Table 6:** IC₅₀ values (µg/mL for plant extracts and µM for mitomycin-C) resulting from cytotoxic activities on MCF-7 and Mahlavu cells

Incubation	Cell line	Treatment	IC₅₀ (Limits)	Slope ± Standard error of the mean (Limits)
24 h	Mahlavu	DME	587.58 (455.74–789.54)	0.68 ± 0.03 (0.62–0.74)
		DWE	652.12 (520.96–845.50)	0.85 ± 0.03 (0.77–0.92)
		LME	98.22 (86.36–112.77)	0.81 ± 0.02 (0.75–0.87)
		LWE	210.08 (173.42–260.90)	0.66 ± 0.02 (0.60–0.71)
		Mitomycin-C	90.94 (78.53–106.73)	0.86 ± 0.03 (0.80–0.92)
	MCF-7	DME	131.22 (115.75–150.23)	0.91 ± 0.03 (0.85–0.97)
		DWE	990.33 (704.30–1492.08)	0.57 ± 0.03 (0.51–0.63)
		LME	34.48 (29.59–40.35)	0.57 ± 0.02 (0.52–0.62)
		LWE	114.74 (92.79–145.99)	0.49 ± 0.02 (0.44–0.54)
		Mitomycin-C	39.61 (35.40–44.58)	0.93 ± 0.02 (0.88–0.99)
48 h	Mahlavu	DME	162.73 (143.11–187.08)	0.95 ± 0.03 (0.88–1.01)
		DWE	175.79 (147.92–213.07)	0.70 ± 0.02 (0.64–0.75)
		LME	24.44 (21.79–27.41)	0.78 ± 0.02 (0.73–0.84)
		LWE	25.26 (22.85–27.90)	0.92 ± 0.02 (0.87–0.98)
		Mitomycin-C	20.05 (17.79–22.69)	0.78 ± 0.02 (0.73–0.83)
	MCF-7	DME	53.65 (48.80–59.17)	1.03 ± 0.03 (0.97–1.09)
		DWE	254.91 (204.86–327.46)	0.60 ± 0.02 (0.55–0.66)
		LME	9.24 (7.47–11.21)	0.48 ± 0.02 (0.43–0.53)
		LWE	28.50 (23.95–33.97)	0.49 ± 0.02 (0.45–0.54)
		Mitomycin-C	9.62 (8.66–10.70)	0.87 ± 0.02 (0.82–0.93)
72 h	Mahlavu	DME	42.71 (39.32–46.47)	1.19 ± 0.03 (1.12–1.25)
		DWE	48.52 (43.42–54.45)	0.84 ± 0.02 (0.79–0.90)
		LME	7.01 (6.16–7.91)	0.86 ± 0.02 (0.81–0.92)
		LWE	8.48 (7.82–9.17)	1.42 ± 0.03 (1.35–1.49)
		Mitomycin-C	2.21 (1.95–2.49)	0.90 ± 0.02 (0.84–0.95)
	MCF-7	DME	36.01 (33.19–39.11)	1.20 ± 0.03 (1.14–1.27)
		DWE	122.63 (100.99–152.50)	0.55 ± 0.02 (0.50–0.60)
		LME	0.66 (0.35–1.08)	0.36 ± 0.02 (0.31–0.41)
		LWE	4.12 (3.47–4.83)	0.74 ± 0.02 (0.69–0.80)
		Mitomycin-C	0.79 (0.67–0.91)	0.98 ± 0.03 (0.92–1.05)

DME: Methanol extract of *D. elegans* var. *actinopetalus*; DWE: Water extract of *D. elegans* var. *actinopetalus*; LME: Methanol extract of *L. ciliatum*; LWE: Water extract of *L. ciliatum*

In both cell lines, the effects of the extracts increased with concentration and time, but the intensity of cytotoxicity varied depending on cell type. In Mahlavu cells, DME and DWE extracts caused a pronounced reduction in viability earlier (at 48 h) at higher concentrations, whereas in MCF-7 cells, the reduction was more gradual, reaching the lowest values at 72 h. Moreover, although LWE and LME extracts exhibited viability rates close to the control at lower concentrations in both cell lines, a more rapid decline was observed in Mahlavu cells during prolonged treatments. These results suggest that Mahlavu cells are more sensitive to the extracts, while MCF-7 cells display greater resistance.

### Antibacterial activity

The antimicrobial effects of methanol and water extracts obtained from the plant samples were evaluated against *S. aureus*, *B. cereus*, *E. coli*, and *P. aeruginosa* strains using the disk diffusion method. Disks impregnated with extracts at a concentration of 40 mg/mL and a volume of 20 µL were compared with standard antibiotic disks (gentamicin, tetracycline, and penicillin) used as positive controls, and solvent-impregnated disks used as negative controls. After the incubation period, the diameters of the inhibition zones formed around the disks were measured, and the obtained data are presented in Table 7.

**Table 7:** Inhibition zones related to the antimicrobial activity of plant extracts

Microorganism	Inhibition area (mm)						
	DME	DWE	LME	LWE	Gentamicin (10 mcg)	Penicillin (10 U)	Tetracycline (30 mcg)
*B. cereus*	nd	nd	nd	nd	24 ± 0	9 ± 0	35 ± 0
*E. coli*	nd	nd	nd	nd	11 ± 1	12 ± 0	24 ± 2
*S. aureus*	nd	nd	nd	nd	16 ± 1	42 ± 2	34 ± 2
*P. aeruginosa*	nd	nd	nd	nd	20 ± 1	-	17 ± 1

Each value is expressed as mean ± standard deviation (*n* = 3). nd: not determined. DME: Methanol extract of *D. elegans* var. *actinopetalus*; DWE: Water extract of *D. elegans* var. *actinopetalus*; LME: Methanol extract of *L. ciliatum*; LWE: Water extract of *L. ciliatum*

In the antimicrobial activity tests performed by the disk diffusion method, it was determined that the evaluated plant extracts did not produce any inhibition zones against *S. aureus*, *B. cereus*, *E. coli*, and *P. aeruginosa* bacteria. In addition, no inhibition zones were observed in the solvent control groups during the experiments.

In contrast, the antibiotics used as positive controls produced distinct inhibition zones against the tested strains. In particular, tetracycline showed high activity against all bacteria, exhibiting the strongest effect with inhibition zones reaching up to 35 mm. Gentamicin also provided significant inhibition against all bacteria, with inhibition zone diameters ranging between 11 and 24 mm. Penicillin displayed variable activity depending on the bacterial species, showing the highest inhibition against *S. aureus* (42 mm), limited effect against *B. cereus* and *E. coli*, and no inhibition against *P. aeruginosa*. These results indicate that the plant extracts used in this study did not exhibit antimicrobial activity at the tested concentration, whereas the control antibiotics confirmed their expected efficacy profiles.

To evaluate the antimicrobial activities of the plant extracts, MIC was determined against *S. aureus*, *B. cereus*, *E. coli*, and *P. aeruginosa* strains. For this purpose, extracts prepared at different concentrations (0–40 mg/mL) were mixed with the test bacteria in 96-well microplates, and bacterial growth was visually assessed after incubation at 37 °C for approximately 16 h. In addition to the extracts, ampicillin and streptomycin were used as standard antibiotics for comparative evaluation, and MIC values were determined for each bacterial strain. The results obtained are presented in Table 8.

**Table 8:** MIC values ​​obtained with plant extracts and standard antibiotics

Microorganism	MIC value (µg/mL)					
	DME	DWE	LME	LWE	Ampisilin	Streptomisin
*B. cereus*	nd	nd	nd	nd	250	15.625
*E. coli*	nd	nd	nd	nd	62.5	15.625
*S. aureus*	nd	nd	nd	nd	7.8125	31.25
*P. aeruginosa*	nd	nd	nd	nd	> 500	31.25

nd: not determined. DME: Methanol extract of *D. elegans* var. *actinopetalus*; DWE: Water extract of *D. elegans* var. *actinopetalus*; LME: Methanol extract of *L. ciliatum*; LWE: Water extract of *L. ciliatum*

The inhibitory effects of the plant extracts on the growth of the tested microorganisms were evaluated, and the findings indicated that none of the extracts reached a detectable MIC value within the examined concentration range (0–40 mg/mL). This outcome confirms that the extracts did not exhibit significant antibacterial activity against the tested Gram-positive (*S. aureus*, *B. cereus*) and Gram-negative (*E. coli*, *P. aeruginosa*) bacteria, consistent with the results obtained from the disk diffusion analyses.

In contrast, the antibiotics used as positive controls demonstrated inhibitory effects on the growth of the test microorganisms. Ampicillin showed strong activity with low MIC values, particularly against *S. aureus* (7.8125 µg/mL) and *E. coli* (62.5 µg/mL). Streptomycin exhibited a broader spectrum of activity, with the lowest MIC values observed against *B. cereus* and *E. coli* (15.625 µg/mL). These findings confirm the validity of the antibiotics as reference standards, while indicating that the tested plant extracts did not display notable antibacterial potential under the experimental conditions.

### Heatmap, hierarchical clustering, and 3D density analyses

The heatmap generated from the correlation analysis provided a detailed visualization of the direction and strength of the relationships among phenolic acids, flavonoids, antioxidant activities, and cytotoxic activities. In the applied color scale, red tones indicate strong positive correlations, whereas green tones represent negative correlations between variables. Overall, the analysis revealed that a substantial proportion of the variables exhibited strong positive associations, as reflected by the predominance of intense red regions. Nevertheless, the presence of distinct negative correlations (green regions) between certain parameters suggests that the extract treatments may exert differential or opposing effects on specific biological activities. This observation indicates that the bioactive compounds present in plant extracts do not act through a unidirectional mechanism but rather through complex, multidimensional, and interactive modes of action.

The dendrogram analysis further demonstrated that variables clustered according to similarities in their correlation patterns, resulting in the formation of distinct groups. Four major clusters were identified, among which protocatechuic acid formed a separate cluster, indicating a unique and independent effect profile compared with other phenolic acids. Moreover, examination of the relationships between antioxidant activities (DPPH radical scavenging and metal chelating activity) and phenolic acid–flavonoid compounds revealed that *p*-coumaric acid, chlorogenic acid, isoferulic acid, and resveratrol clustered closely together, displaying strong positive correlations. This clustering pattern supports the notion that these compounds contribute substantially to the overall antioxidant capacity of the extracts.

**Fig. 6:**
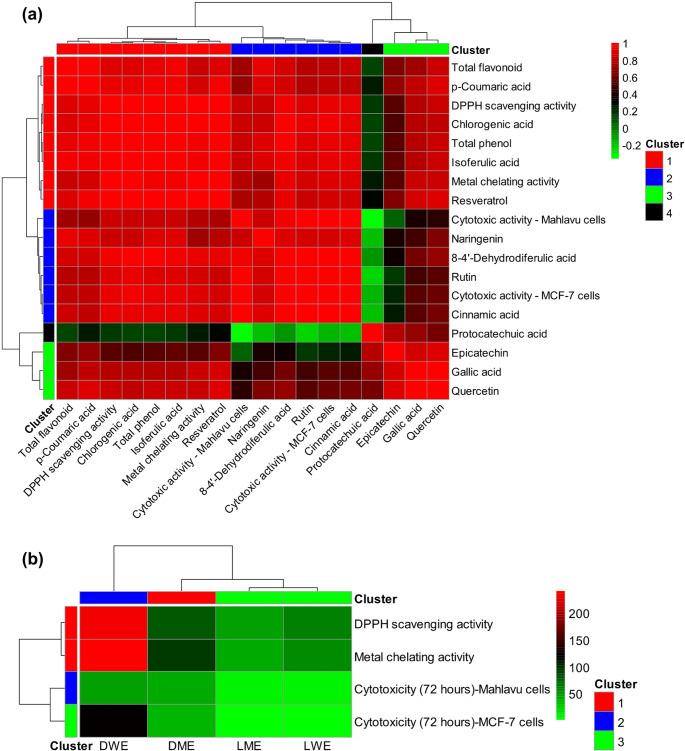
**a** Representation of the correlations among the antioxidant and cytotoxic activities, total phenolic content, total flavonoid content, and phenolic compounds of the extracts using heat map and clustering analyses. **b** Heat map and clustering analyses based on the IC₅₀ values of the extracts for their antioxidant and cytotoxic activities. DME: Methanol extract of *D. elegans* var. *actinopetalus*; DWE: Water extract of *D. elegans* var. *actinopetalus*; LME: Methanol extract of *L. ciliatum*; LWE: Water extract of *L. ciliatum*

In terms of cytotoxic activities, the effects observed on Mahlavu and MCF-7 cells were found to be associated with naringenin, 8 − 4’-dehydrodiferulic acid, rutin, and cinnamic acid. This result highlights the potential anticancer significance of these phenolic compounds. Additionally, the positive correlation observed between total phenolic and flavonoid contents and antioxidant activities provides important evidence supporting the central role of these compounds in the biological activity of plant extracts (Fig. 6a).

Based on the IC₅₀ values calculated from the antioxidant and cytotoxic activities of the extracts, the heat map and clustering analyses clearly revealed distinct effect profiles among the extracts. Three main clusters were identified in these analyses. LME and LWE, which exhibited the lowest IC₅₀ values across all activities, were grouped together, while DME and DWE formed separate clusters. Notably, DWE, characterized by generally high IC₅₀ values, stood out as the extract with the weakest biological activity overall (Fig. 6b).

**Fig. 7:**
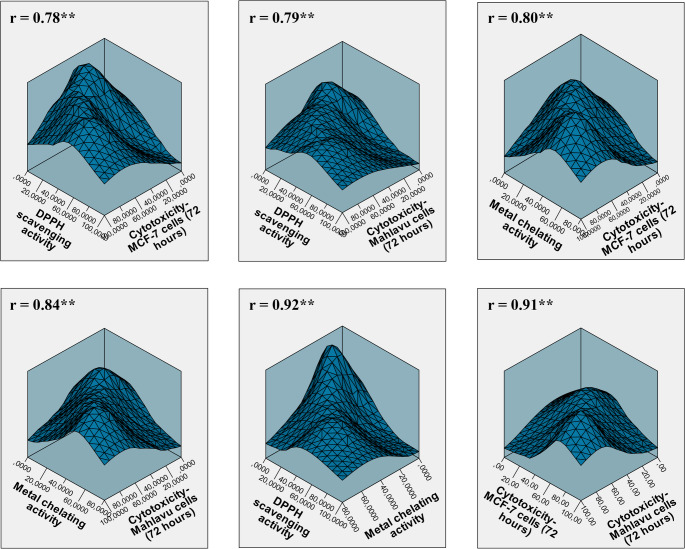
3D density analysis created to assess the relationships among the DPPH radical scavenging, metal chelating, and cytotoxic activities of the extracts. The calculated correlation coefficients (r) indicate statistical significance at the *p* < 0.01 level

In the 3D surface plots illustrating the relationship between the antioxidant activities and cytotoxic effects of the plant extracts, strong and significant positive correlations were observed among all parameters (*r* = 0.78–0.92, *p* < 0.01). A strong relationship was found between DPPH radical scavenging activity and cytotoxicity in both MCF-7 (*r* = 0.78) and Mahlavu cells (*r* = 0.79), indicating that extracts with higher free radical scavenging capacity were also more effective in suppressing cancer cell proliferation. Similarly, significant correlations were observed between metal chelation activity and cytotoxicity (*r* = 0.80–0.84), suggesting that the ability to bind metal ions may influence mechanisms related to cell death. The high correlation level between DPPH activity and cytotoxicity further indicates that free radical scavenging capacity is a major determinant of cytotoxic potential. Overall, these findings demonstrate that the antioxidant potential and anticancer activities of the extracts are closely interrelated (Fig. 7).

### Molecular docking analysis

To further elucidate the molecular basis of the observed cytotoxic effects, an *in silico* molecular docking analysis was performed between selected phenolic compounds and the anti-apoptotic Bcl-2 protein. The phenolic compounds subjected to docking were chosen based on two main criteria: (i) their relatively high concentrations identified by HPLC analysis in the methanol extracts, particularly in *L. ciliatum*, and (ii) their prominent biological relevance reported in cancer-related studies. Accordingly, chlorogenic acid, quercetin, and naringenin—representing the most abundant and biologically significant phenolics—were selected for detailed interaction analysis, while additional phenolic compounds were included for comparative evaluation.

Bcl-2 was selected as the target protein due to its pivotal role in the regulation of apoptosis and its overexpression in various cancer types, including breast and liver cancers. The inhibition of Bcl-2 is known to promote apoptotic cell death by disrupting mitochondrial membrane integrity. Therefore, the strong binding interactions observed between Bcl-2 and the selected phenolic compounds may partially explain the pronounced cytotoxic effects of the phenolic-rich methanol extracts, particularly against MCF-7 and Mahlavu cancer cell lines.

**Fig. 8:**
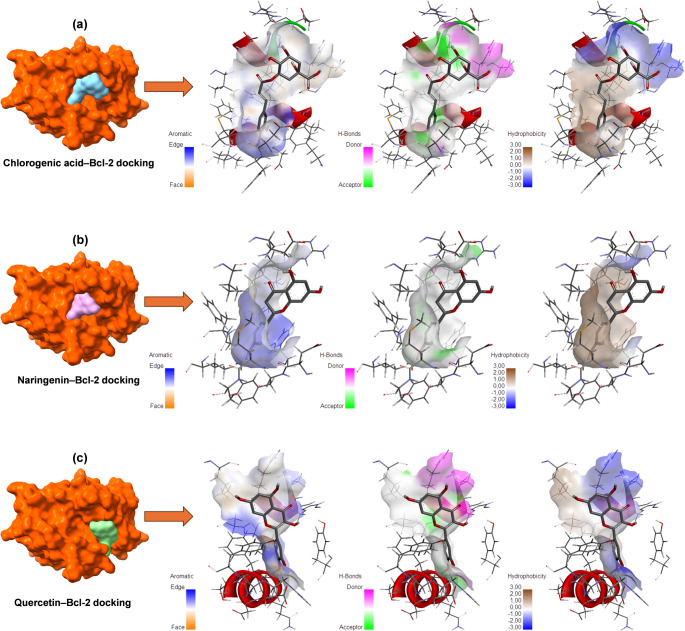
*In silico* molecular docking analysis of Bcl-2 protein with **a** chlorogenic acid, **b** naringenin, and **c** quercetin. The left panels show the surface representation of Bcl-2, with the ligand-binding pocket highlighted. The middle panels illustrate the docked conformations of each ligand within the Bcl-2 binding groove. The right panels present molecular interaction surface maps, including aromatic interaction regions (edge and face), hydrogen bond donor and acceptor sites, and hydrophobicity distributions. Color gradients indicate aromatic edge (blue) and face (orange) interactions, hydrogen bond donors (magenta) and acceptors (green), and hydrophobic (brown) to hydrophilic (blue) regions. These representations collectively demonstrate the complementary electrostatic, hydrophobic, and aromatic interactions contributing to ligand stabilization within the anti-apoptotic Bcl-2 binding pocket

Docking results demonstrated that all tested phenolic compounds exhibited favorable binding affinities toward the Bcl-2 protein, with binding energies ranging from − 5.4 to − 7.3 kcal/mol. Among them, chlorogenic acid showed the strongest binding affinity (− 7.3 kcal/mol), followed by quercetin (− 7.2 kcal/mol) and naringenin (− 7.0 kcal/mol) (Table 9). These three compounds also correspond to the phenolics detected at the highest levels in the extracts, suggesting a direct relationship between phenolic abundance and potential biological activity. The remaining compounds, including 8 − 4′-dehydrodiferulic acid and resveratrol, displayed moderate binding affinities, whereas gallic acid exhibited comparatively weaker interaction with Bcl-2. The RMSD values of 0.00 for all docked complexes indicate stable and reliable binding conformations. Molecular docking analysis revealed that chlorogenic acid, naringenin, and quercetin were all successfully accommodated within the binding groove of the Bcl-2 protein (Fig. 8). Surface representations indicated that all three ligands occupied a similar hydrophobic cavity, suggesting a common binding region within the Bcl-2 structure.

**Table 9:** *In *silico molecular docking results showing the binding affinities of major phenolic compounds toward the Bcl-2 protein

Ligand	Binding affinity (kcal/mol)	RMSD lower bound	RMSD upper bound
8 − 4′-Dehydrodiferulic acid (C_20_H_18_O_8__ID.157009740)	-6.9	0.00	0.00
Chlorogenic acid (C_16_H_18_O_9__ID.1794427)	-7.3	0.00	0.00
Gallic acid (C_7_H_6_O_5__ID.370)	-5.4	0.00	0.00
Naringenin (C_15_H_12_O_5__ID.439246)	-7.0	0.00	0.00
Quercetin (C_15_H_10_O_7__ID.5280343)	-7.2	0.00	0.00
Resveratrol (C_14_H_12_O_3__ID.445154)	-6.7	0.00	0.00

Chlorogenic acid exhibited an extended binding conformation within the Bcl-2 pocket, forming multiple stabilizing interactions. Interaction surface maps demonstrated the presence of both hydrogen bond donor and acceptor regions surrounding the ligand, along with aromatic interaction features that contributed to ligand positioning. Hydrophobicity mapping further indicated a favorable complementarity between the ligand and the surrounding binding site residues (Fig. 8a).

Naringenin adopted a comparatively compact orientation within the binding pocket. Aromatic interaction surfaces were observed predominantly along one side of the ligand, while hydrogen bond donor and acceptor regions were more limited in distribution compared to chlorogenic acid. The hydrophobicity surface suggested moderate hydrophobic complementarity, indicating stable but relatively fewer interaction points within the binding cavity (Fig. 8b).

Quercetin displayed a distinct binding mode characterized by pronounced aromatic and hydrogen bonding features. Interaction maps showed extensive aromatic face and edge interactions, as well as clearly defined hydrogen bond donor and acceptor regions aligned with the ligand’s polyphenolic structure. Hydrophobicity analysis revealed a balanced distribution of hydrophobic and hydrophilic regions, supporting effective accommodation of quercetin within the Bcl-2 binding groove (Fig. 8c). Overall, the docking visualizations demonstrated that all three polyphenolic compounds interact with Bcl-2 through a combination of aromatic, hydrogen bonding, and hydrophobic interactions, with differences in interaction patterns and surface complementarity observed among the ligands.

**Fig. 9:**
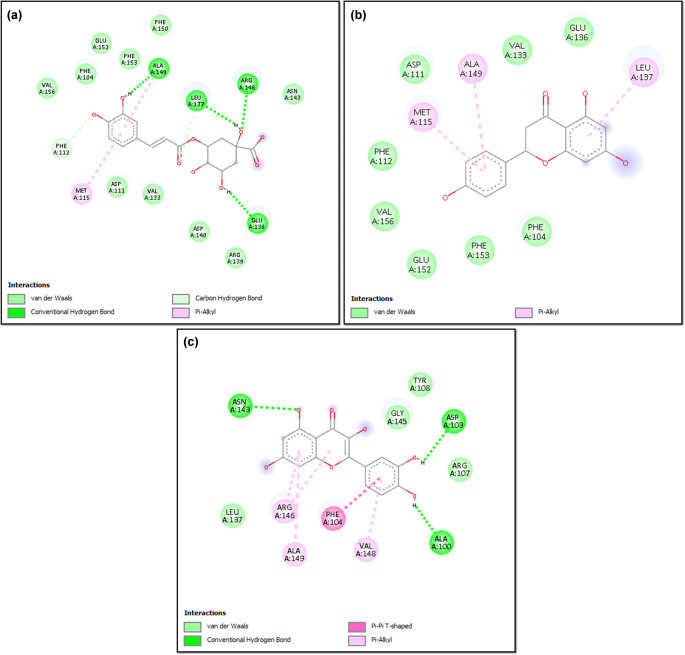
Two-dimensional (2D) ligand–protein interaction diagrams illustrating the binding modes of Bcl-2 with **a** chlorogenic acid, **b** naringenin, and **c** quercetin. The figures depict key amino acid residues involved in ligand stabilization within the Bcl-2 binding pocket. Green dashed lines indicate conventional hydrogen bonds, light green highlights represent van der Waals interactions, while pink dashed lines denote π–alkyl and π–π stacked interactions. These non-covalent interactions collectively contribute to the binding affinity and structural stabilization of the ligands within the anti-apoptotic Bcl-2 protein

Two-dimensional (2D) interaction analysis showed that chlorogenic acid, naringenin, and quercetin bind to the Bcl-2 protein through non-covalent interactions involving specific amino acid residues within the binding pocket (Fig. 9). Chlorogenic acid and quercetin formed multiple conventional hydrogen bonds and aromatic interactions with residues such as ALA149, ARG146, ASP103, GLU136, and PHE104, whereas naringenin primarily interacted with Bcl-2 via van der Waals and π–alkyl interactions involving residues including PHE104, PHE112, MET115, and VAL156. Overall, the interaction patterns indicated a higher diversity of binding interactions for chlorogenic acid and quercetin compared to naringenin.

## Discussion

In this study, the phenolic/flavonoid contents, antioxidant activities, cytotoxic and antimicrobial effects of methanol and aqueous extracts obtained from the endemic species *D. elegans* var. *actinopetalus* and *L. ciliatum* were comprehensively investigated. The absence of previous studies on the biological activities of these two species in the literature increases the scientific originality of the findings obtained. However, various biological activities of different species belonging to the same genera have been previously reported (Boukeria et al. 2020; Mkedder et al. 2021; Baqer et al. 2023). Therefore, this study not only provides new contributions but also presents findings that support the existing literature.

As a result of the HPLC analyses, LME was identified as the richest extract in terms of phenolic and flavonoid compounds. The predominance of bioactive compounds such as chlorogenic acid, gallic acid, cinnamic acid, p-coumaric acid, and quercetin explains the strong pharmacological potential of this extract. Phenolic and flavonoid compounds exert their effects through mechanisms such as reducing oxidative stress, scavenging free radicals, chelating metal ions, and regulating intracellular signaling pathways (Chaudhary et al. 2023b; Sadiq 2023; Rao and Zheng 2025). In particular, compounds such as quercetin and gallic acid are known to possess both strong antioxidant and anticancer properties (Sachithanandam et al. 2022; Azeem et al. 2023; Keyvani-Ghamsari et al. 2023). Therefore, the parallelism observed between the obtained phenolic/flavonoid profile and biological activities is remarkable.

The higher phenolic and flavonoid contents in methanol extracts compared to aqueous extracts are due to differences in solvent polarity (Abdelbaky 2021; Chatepa et al. 2024). The polarity of the solvent directly affects the solubility and extraction efficiency of biologically active compounds from the plant matrix. Similarly, previous studies on the *Dianthus* (Saboora et al. 2013; Wang et al. 2024; Farajzadeh‑Memari‑Tabrizi et al. 2025) and *Linum* (Alachaher et al. 2018; Esmaeili et al. 2023; Koçak 2024) genera have reported that methanol extracts contain higher levels of phenolic compounds compared to aqueous extracts. This can be explained by the strong interaction of methanol with hydroxyl groups in phenolic structures, facilitating the extraction of these compounds.

In the present study, the highest activities in both DPPH radical scavenging and metal chelating assays were observed in LME, a finding that is directly attributable to its high phenolic and flavonoid content. The antioxidant efficacy of phenolic compounds is well established and is largely dependent on the radical-scavenging capacity of hydroxyl groups within their chemical structures. Notably, chlorogenic acid and quercetin have been identified as potent antioxidants capable of effectively neutralizing free radicals, thereby markedly reducing cellular oxidative damage (Sadeghi et al. 2023; Lian and Yang 2025). Accordingly, the high antioxidant capacity observed in LME demonstrates that the diversity and concentration of its phenolic compounds are strongly associated with its biological effects.

The findings obtained are largely consistent with previous studies on *Linum* and *Dianthus* species. Especially *Linum* extracts have been reported in several studies to be rich in phenolic compounds and, therefore, to exhibit significant antioxidant activity (Hasiewicz-Derkacz et al. 2015; Gai et al. 2023; Ghozzi et al. 2024). The diversity and concentration of phenolic compounds enhance the ability of *Linum* extracts to effectively neutralize free radicals and support their biological activities. Similarly, studies on *Dianthus* species have revealed a strong positive relationship between phenolic content and antioxidant capacity (Yu et al. 2007; Saboora et al. 2013; Bashir et al. 2024). These results indicate that the chemical composition of extracts plays a critical role in determining biological activity across different plant genera.

The findings of this study strongly indicate that the elevated phenolic and flavonoid content of LME is directly associated with its pronounced antioxidant activity. In this regard, LME may be considered a promising natural source of antioxidants with potential to alleviate oxidative stress in biological systems. Moreover, phenolic-rich extracts have been reported to offer benefits beyond pharmaceutical applications, extending to the development of functional foods (Panda et al. 2025; Rutkowska and Pasqualone 2025). Therefore, LME appears to have strong potential for use as a natural antioxidant additive in the pharmaceutical, nutraceutical, and food industries.

Cytotoxicity tests conducted on Mahlavu (liver cancer) and MCF-7 (breast cancer) cell lines revealed that LME and LWE extracts exhibited remarkable antiproliferative effects. In particular, the IC₅₀ value of 0.66 µg/mL obtained for LME after 72 h in MCF-7 cells indicates a strong anticancer potential. In the literature, lignans and phenolic compounds of *Linum* species have been reported to induce apoptosis, arrest the cell cycle, and suppress metastasis in breast cancer cells (Szewczyk et al. 2014; Mouna et al. 2022). Similarly, extracts of *Dianthus* species have been reported to possess cytotoxic effects in various cancer cell lines, including colon (Martineti et al. 2010), liver (Yu et al. 2012), and breast (Hamad et al. 2024) cancers.

In our study, the higher cytotoxic activity of methanol extracts appears to be related to their phenolic/flavonoid richness. Moreover, the increase in cytotoxic effect with prolonged incubation time suggests that the biological activity of the extracts strengthens over time, which may be associated with intracellular metabolism and the bioavailability of extract compounds (Choudhury et al. 2014; Chen et al. 2023a).

These results were further supported by correlation analyses and heatmap findings. Examination of the relationships among phenolic acids, flavonoids, antioxidant activities, and cytotoxic activities revealed predominantly high positive correlations. In particular, the significant correlations between DPPH radical scavenging and metal chelating activities with cytotoxicity in both MCF-7 and Mahlavu cells (*r* = 0.78–0.84, *p* < 0.01) demonstrate that free radical scavenging and metal ion binding capacities directly contribute to anticancer efficacy. Indeed, several studies have shown that phenolic compounds regulate ROS levels, activate mitochondrial pathways, trigger caspase cascades, and induce apoptosis through DNA damage (Olivas-Aguirre et al. 2020; Do et al. 2021; Zhao et al. 2023b). The findings of this study revealed that phenolics such as p-coumaric acid, chlorogenic acid, isoferulic acid, and resveratrol were highly associated with antioxidant capacity, while compounds such as naringenin, rutin, and cinnamic acid were linked to cytotoxic effects. In conclusion, the results demonstrate that both antioxidant and cytotoxic activities are directly related to phenolic/flavonoid concentration, with LME emerging as the most prominent extract in this regard. This suggests that *Linum* extracts may be evaluated as natural anticancer and antioxidant agents for potential pharmaceutical and nutraceutical applications.

The antimicrobial activities of the methanolic and aqueous plant extracts were assessed using the disk diffusion and MIC assays. The results demonstrated that neither extract exhibited inhibitory effects against the tested Gram-positive bacteria (*S. aureus* and *B. cereus*) or Gram-negative bacteria (*E. coli* and *P. aeruginosa*) within the concentration range examined. In contrast, the positive control antibiotics—ampicillin, streptomycin, tetracycline, and gentamicin—produced pronounced antibacterial effects, as expected. The lack of antibacterial activity observed for the plant extracts may be attributed to insufficient concentrations of bioactive constituents capable of disrupting bacterial cell wall integrity or interfering with essential metabolic pathways. Consistent with this observation, numerous plant species have been reported to exhibit strong antioxidant or cytotoxic activities while displaying limited or negligible antibacterial effects (Ge et al. 2019; Karahüseyin et al. 2024; Taghizadeh and Jalili 2024). It is well known that the lipopolysaccharide membrane structure of Gram-negative bacteria confers resistance by preventing phenolic compounds from entering the cell (Breijyeh et al. 2020; Saxena et al. 2023). Another reason for this difference lies in the structural distinctions between prokaryotic and eukaryotic cells. Bacteria (prokaryotic cells) are simple structures lacking nuclei and organelles, possessing rigid cell walls that act as barriers against many bioactive compounds. In contrast, cancer cells, which are eukaryotic in nature, have complex biochemical processes involving nuclei and organelle systems. Therefore, while phenolic compounds in extracts may be ineffective against bacteria, they can induce apoptosis and oxidative stress–related cytotoxic effects in cancer cells (Oršolić and Jazvinšćak Jembrek 2022; Chimento et al. 2023).

Nevertheless, the same extracts exhibited strong antioxidant properties and cytotoxic effects on breast and liver cancer cells at a concentration of 400 µg/mL. This indicates that the biological activities of the extracts are primarily concentrated on free radical scavenging and inhibition of cancer cell growth rather than antimicrobial effects. Similarly, the literature reports that some plant extracts show limited antimicrobial activity but pronounced oxidative stress modulation and anticancer effects (Shan et al. 2024; Al-Rashidi et al. 2025). The findings obtained suggest that these extracts cannot serve as direct antibiotic alternatives but can be evaluated as antioxidant and anticancer agents in pharmaceutical and nutraceutical applications. Moreover, the combination of plant extracts with antibiotics may enhance antimicrobial activity through synergistic effects (Vaou et al. 2022; Soulaimani 2025).

The molecular docking analysis provided mechanistic insights into the cytotoxic effects observed for the phenolic-rich extracts, particularly LME. The anti-apoptotic protein Bcl-2 was selected as the target due to its pivotal role in regulating cell survival and apoptosis. Overexpression of Bcl-2 is commonly observed in multiple cancer types, including breast (MCF-7) and liver (Mahlavu) cancer cells, and contributes to chemoresistance by inhibiting mitochondrial-mediated apoptotic pathways (Bahar et al. 2022; Shenoy and Abdul Salam 2025). Therefore, targeting Bcl-2 provides a strategic approach to induce apoptosis in cancer cells.

Docking results revealed that the major phenolic compounds—chlorogenic acid, quercetin, and naringenin—exhibited strong binding affinities toward Bcl-2, suggesting potential interference with its anti-apoptotic function. Notably, these compounds were also among the most abundant in LME, supporting a correlation between phenolic content and cytotoxic activity, consistent with previous reports on polyphenol-mediated apoptosis (Benvenuto et al. 2020). Although docking provides predictive insights into ligand–protein interactions, it complements rather than replaces *in vitro* cytotoxicity assays, reinforcing the hypothesis that the anticancer effects of LME phenolics may, at least in part, be mediated through modulation of Bcl-2 activity, thereby promoting apoptotic cell death in Mahlavu and MCF-7 cancer cell lines.

## Conclusions

In this study, the phenolic and flavonoid contents, antioxidant activities, as well as the cytotoxic and antimicrobial effects of methanol and aqueous extracts from the endemic species *D. elegans* var. *actinopetalus* and *L. ciliatum* were comprehensively investigated and supported by *in silico* molecular docking analysis. The lack of previous reports on the biological activities of these endemic plants underscores the originality and scientific relevance of the present study.

The results demonstrated that the LME contained the highest levels of phenolic and flavonoid compounds, which were strongly correlated with its pronounced antioxidant and cytotoxic activities. HPLC analysis identified chlorogenic acid, gallic acid, cinnamic acid, *p*-coumaric acid, and quercetin as the major bioactive constituents. These phenolic compounds are well known to exert their biological effects through multiple mechanisms, including free radical scavenging, metal ion chelation, attenuation of oxidative stress, and modulation of intracellular signaling pathways. Notably, the remarkably low IC₅₀ values observed in MCF-7 breast cancer cells indicate a potent anticancer potential of LME.

To further elucidate the molecular mechanisms underlying the observed cytotoxic effects, *in silico* molecular docking analysis was performed using the anti-apoptotic protein Bcl-2 as a target. The docking results revealed that the most abundant phenolic compounds, particularly chlorogenic acid, quercetin, and naringenin, exhibited strong binding affinities toward Bcl-2. These findings suggest that the anticancer activity of phenolic-rich extracts may be partially mediated through the modulation of apoptosis-related pathways via interactions with Bcl-2, thereby supporting the *in vitro* cytotoxicity findings.

In contrast, neither of the plant extracts exhibited notable antibacterial activity against the tested Gram-positive or Gram-negative bacterial strains. This observation indicates that the biological effects of the extracts are predominantly directed toward antioxidant defense and inhibition of cancer cell proliferation rather than antimicrobial activity. Similar findings have been reported in previous studies, where phenolic-rich plant extracts display strong antioxidant and anticancer properties but limited antibacterial efficacy, possibly due to restricted penetration of phenolic compounds through bacterial cell walls and outer membrane structures.

Overall, these results suggest that *L. ciliatum* methanol extract possesses considerable potential as a natural antioxidant and anticancer agent for pharmaceutical and nutraceutical applications. Furthermore, the integration of *in vitro* and *in silico* evidence highlights its promise as a functional bioactive ingredient for the development of functional foods and natural health products.

## Data Availability

No datasets were generated or analysed during the current study.
